# Lack of Efficacy of a Salience Nudge for Substituting Selection of Lower-Calorie for Higher-Calorie Milk in the Work Place

**DOI:** 10.3390/nu7064336

**Published:** 2015-06-02

**Authors:** Amy L. Wilson, Svetlana Bogomolova, Jonathan D. Buckley

**Affiliations:** 1 Ehrenberg-Bass Institute for Marketing Science, University of South Australia, Adelaide 5000, Australia; E-Mail: Svetlana@MarketingScience.Info; 2 Alliance for Research in Exercise, Nutrition and Activity (ARENA), Sansom Institute for Health Research, University of South Australia, Adelaide 5000, Australia; E-Mail: Jon.Buckley@unisa.edu.au

**Keywords:** nudging, behavioural economics, salience nudge, obesity, milk selection, workplace

## Abstract

Obesity is a major burden on healthcare systems. Simple, cost effective interventions that encourage healthier behaviours are required. The present study evaluated the efficacy of a salience nudge for promoting a change in milk selection from full-cream to low-fat (lower-calorie) in the kitchen of a university-based research institute that provided full-cream and low-fat milk free of charge. Milk selection was recorded for 12 weeks (baseline). A sign with the message “*Pick me! I am low calorie*” was then placed on the low-fat milk and selection was recorded for a further 12 weeks. During baseline, selection of low-fat milk was greater than selection of full-cream milk (*p* = 0.001) with no significant milk-type × time interaction (*p =* 0.12). During the intervention period overall milk selection was not different from baseline (*p =* 0.22), with low-fat milk selection remaining greater than full-cream milk selection (*p* < 0.001) and no significant milk-type × time interaction (*p =* 0.41). However, sub-analysis of the first two weeks of the intervention period indicated an increase in selection of both milk types (*p =* 0.03), but with a greater increase in low-fat milk selection (*p =* 0.01, milk-type × time interaction). However, milk selection then returned towards baseline during the rest of the intervention period. Thus, in the present setting, salience nudging promoted a transient increase in low-fat milk selection, but also increased selection of full-cream milk, indicating that nudging was not effective in promoting healthier milk choices.

## 1. Introduction

Overweight and obesity occur when energy intake exceeds energy expenditure over time [1,2]. The majority of short-term weight loss strategies reduce energy intake through calorie-controlled diets that can require substantial changes to the routine of individuals [3,4]. However, long-term dieting adherence can be difficult to achieve [4,5,6]. The Dual Process model of information processing [7,8] may explain some of the difficulty people have in maintaining healthy dietary changes. The Dual Process model proposes that information processing comprises rational and intuitive components. Formation of new healthy behaviours is an intentional behaviour change, and is proposed to be governed by the rational system which is usually slow, logical and deliberate. However, intentional change may be compromised by stress, fatigue, emotions, multi-tasking, cognitive load, and other competing intentions, such as taste preferences, satisfying a craving for unhealthy food, saving time and money, or consuming unhealthy food and beverages at social events [7,8]. This is when the intuitive system becomes dominant, turning decision-making into an automatic, subconscious process, controlled by habits [7]. Consumers’ inability to identify and recall more than 90% of food-related decisions made throughout the day [9] highlights the mindless, or habitual, nature of dietary behaviours that are driven by the intuitive system [9,10]. The intuitive nature of habits makes them resistant to change.

One of the ways habitual behaviour may be influenced is by modifying the environment within which decisions are made [11,12,13]. A number of models that have been applied to try and achieve change in health behaviours, such as the Social Cognitive Model and the Ecological and Social Ecological Models [14,15,16,17] which consider the influence of the environment. However, evaluations of the role of *habits* and their resistance to change is currently under-researched and underrepresented in the health literature, with most health behaviour change interventions being based on rational decision-making models [14,18,19,20,21,22].

Behavioural economics places particular emphasis on the role of the environment in influencing *habitual* behaviour. The key tool of influence in behavioural economics is the nudge, which is any addition to, or modification of the environment that is designed to change a consumer’s behaviour in a predictable way [12]. Nudges may increase salience of a particular option or behaviour, making it easier to process, and therefore making that option more appealing. To ensure that freedom of choice is maintained, nudges should be avoidable and the original option should be available so that the choice is voluntary [12].

Some studies have shown that nudging can influence food choices. Increased visibility, proximity and convenience of food items has been shown to increase selection [23], while decreased visibility, proximity and convenience reduced selection [24,25]. Nudges come in different forms [26], including salience nudges. Interventions informed by nudging theory have encouraged healthier dietary choices through highlighting nutrition information such as caloric content, traffic light labeling [27,28,29,30,31,32], or descriptive labels that highlight a certain favorable characteristic of the product (for example, “low-fat”) [33,34,35,36]. However these nudging interventions have been conducted predominantly in cafeterias (mainly hospital settings), and only acute effects have been evaluated, limiting their generalizability. Furthermore their efficacy has been variable, possibly due to failure of people to notice/pay attention to the information provided, or a lack of understanding of its meaning [37]. Seymour *et al*. [38] reviewed nutrition information interventions across multiple environments, and concluded they are more effective when conducted in “limited access” sites, such as workplaces. Salience nudges can include simple messages that are easily understood [26] and therefore may overcome limitations of other nutrition information nudges. Extending this body of knowledge, the current study evaluated salience nudging in a real world environment, and over an extended period of time.

The objective of this study was to evaluate the efficacy of using a salience nudge highlighting the low calorie content of low-fat milk, on increasing the selection of low-fat milk in place of full-cream milk selection in a workplace. Milk is a commonly supplied and regularly consumed beverage in workplaces, and an important part of a healthy diet. However, low-fat milk has fewer calories per serve and therefore consumption of low-fat milk may contribute to a reduction in energy intake and obesity. Importantly, the present study evaluated the effect of nudging over the longer-term rather than just a one-off evaluation of an acute effect, thus evaluating its potential to achieve longer-term behavioural change.

## 2. Experimental Section

Milk selection from the fridge of a university-based research institute was monitored over a 24-week period, from May to December 2013. The kitchen was accessible to approximately 70 staff and research students. The number of people accessing the kitchen each day was not recorded. Ethics approval for the study was obtained from the University of South Australia Human Research Ethics Committee prior to commencement.

Two types of milk were used in the study, one was full-cream and the other was low-fat. The full-cream milk (Paul’s full-cream milk, Parmalat, Brisbane, Australia) had an energy content of 2860 kJ/L and a fat content of 38g/L and the low-fat milk (Pauls trim milk, Parmalat, Australia) had an energy content of 1932 kJ/L and a fat content of 13g/L. Full-cream and low-fat milk were always available free of charge to all people who used the kitchen and this was not changed during the study. Milk selection was recorded daily at the same time (9.30 am).

Baseline observations were collected for a 12-week period, after which a 12-week nudging intervention commenced. The intervention involved placing a salience nudging sign on the low-fat milk which read “*Pick me! I am low calorie*”.

Low-fat milk ran out one time during baseline and three times during the intervention period. The selection of both milk types for those days was disregarded and treated as missing data. Spillage was not accounted for, as it was not known whether this occurred or not. However, spillage may have occurred for either milk type. Data were analysed using a repeated measures linear effects mixed model to take account of missing data. Baseline was analysed separately and, because there was no change in milk selection during this period, the average selection was used as the baseline value for analysis of the effect of the intervention. Milk type (*i.e*., full-cream or low-fat) and time (week) were included as repeated measures within the model as the same kitchen users had access to both milk types and measures of milk selection were repeated daily. Statistical significance was set at *p* < 0.05.

## 3. Results

**Figure 1:**
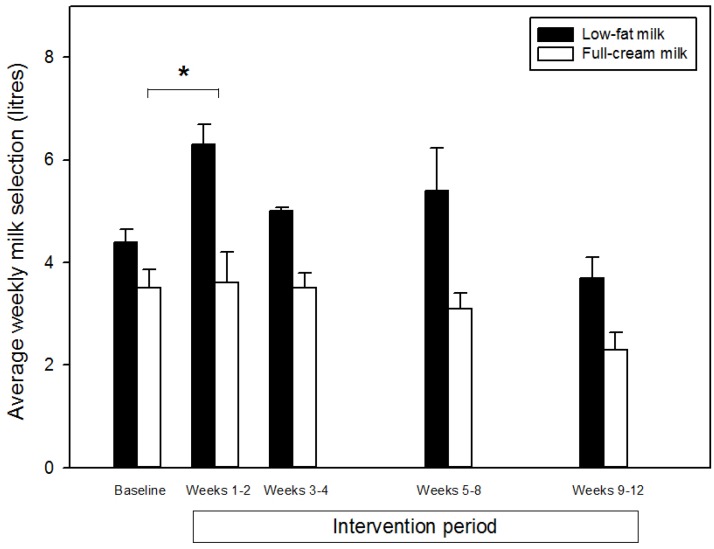
Selection of low-fat and full-cream milk prior to and during a 12 week nudging intervention. Data analysis was based on weekly selection but has been presented as the mean of 12 weeks of baseline data and means of two- and four-weekly periods during the intervention period for simplicity of graphical representation. Data are mean ± standard error. *Significantly different from baseline (*p* = 0.03) and significantly greater increase in low-fat milk selection compared with full-cream milk selection (*p* = 0.01; first two weeks of the intervention period only).

During the baseline period low-fat milk selection was greater than full-cream milk selection (*p =* 0.001), but did not change from week to week (*p =* 0.12, milk-type × time). During the intervention period overall milk selection was not different from baseline (*p =* 0.22), with low-fat milk selection remaining greater than full-cream milk selection (*p* < 0.001) and no significant milk-type × time interaction (*p =* 0.41). However, there appeared to be a transient increase in milk selection during the first part of the intervention period, and sub-analysis of the first two weeks of the intervention period indicated a significant increase in selection of both milk types from baseline (*p =* 0.03), with a significantly greater increase in low-fat milk selection (*p =* 0.01, milk-type × interaction; Figure 1). However, milk selection then returned towards baseline during the rest of the intervention period.

## 4. Discussion

The present study indicates that a salience nudge was not effective in changing milk selection from full-cream to low-fat milk. While sub-analysis did indicate a transiently greater increase in low-fat milk selection during the first two weeks of the intervention, the nudge also increased the selection of full-cream milk during this period, thus increasing total milk selection rather than eliciting a shift from selection of full-cream milk to low-fat milk. Therefore, during the first two weeks of the intervention there was an overall increase in milk selection, meaning that there would have been an increase in caloric intake from milk, which may be detrimental when the purpose of the nudge was to increase selection of low-fat milk in preference to full-cream milk to reduce caloric intake.

The finding of an overall increase in total milk selection, including full-cream milk, during the first two weeks of the intervention period may be due to a spillover effect [39,40] as a result of the nudging sign increasing the salience of milk (*i.e*., increasing awareness of its availability) resulting in an increase in selection of both full-cream and low-fat milk. There is evidence of spillover effects in the marketing literature [41,42], particularly for products that are similar in nature [40], such as was the case for the low-fat and full-cream milk in the present study. Spillover effects have been evident in other nudging interventions, however in those studies overall selection decreased rather than increased. For example, rearranging the environment to increase visibility of healthier items and reduce visibility of less healthy items in a cafeteria decreased purchases of both healthier and less healthy items [32]. Thus, while nudging interventions typically aim to increase selection of healthier items, and decrease selection of less healthy items (*i.e*., substitution of less healthy options with healthier options), this effect does not always occur, but instead changes in overall selection can occur in either direction. The overall lack of efficacy of nudging to change milk selection in the present study is in agreement with the findings of another study by Olstad *et al*. [29] who placed descriptive labels (*i.e*., funky chicken teriyaki wrap) on healthy items, and found no influence on sales. Another study altered placement of snacks across a small set of four shelves to alter visibility of healthy choices and found no influence on snack item selection [43]. As was the case in the present study, these studies altered the visibility of one item, without changing visibility of the other. In contrast, nudging interventions that increased the visibility, accessibility and/or availability of a healthier items while simultaneously decreasing the visibility, accessibility and/or availability of the less healthy items [28,43,44,45,46], have been effective for influencing healthier dietary choices. This suggests that for nudges to be effective in achieving healthier dietary choices, it is not sufficient to only increase availability or awareness of healthier options, but awareness and/or availability of less-healthy options also need to be reduced. Thus, future research could test a message that encourages low-fat milk selection while simultaneously discouraging full-cream milk selection, for example “Pick me (the low-fat option) rather than me (the full-cream milk)!” Another suggestion could be to contrast “Pick me! I am low calorie” placed near the low-fat to “Don’t pick me! I am high calorie” placed near the full-cream milk, to see if positive or negative messaging has different influences on milk selection. The transient effect on milk selection that was observed suggests that while nudges may exhibit efficacy acutely, these effects are short-lived and may subside within a relatively short period of time (*i.e*., within 1–2 weeks), thus future interventions could introduce a new nudging message every two weeks to ensure ongoing salience of the low-fat milk.

The present study had a number of limitations. It is possible that the changes in milk selection behaviour that were observed during the initial phase of the intervention period may have been a result of the Hawthorne effect [47]. This effect occurs when participants are aware that they are being observed, and their behaviour is altered as a result of that knowledge [48]. Participants in the present study did not have their individual behaviour directly observed, so it was not possible to identify whether particular groups of individuals were influenced more than others by the nudge. Nor were people using the kitchen told that research was being conducted. However, as the present study was conducted in the kitchen of a university research institute, the staff may have been suspicious that milk selection was being observed during the intervention period when the sign appeared on the low-fat milk. The fact that the intervention was conducted in the kitchen of a University-based research institute might limit the generalizability of the findings to other settings. Staff and students who use this kitchen are likely to be from a higher socio-demographic and educational status and numerous studies have reported healthier dietary behaviours among higher socio-demographic groups with better education [49,50]. While the health characteristics and dietary intake of the population are unknown, our data indicated that during the baseline period there was greater selection of low-fat milk compared with full-cream milk. This contrasts with figures for overall milk consumption in Australia which show that consumption of full-cream milk is almost double that of low-fat milk (*i.e*., 1169 million litres of full-cream milk per annum *vs.* 687 million litres of low-fat milk per annum) [51]. The greater selection of low-fat milk compared with full-cream milk at baseline in the present study not only suggests that the study sample observed was more health-conscious than the average Australian, and thus not a representative sample, it would also be expected to make it more difficult to further increase low-fat milk selection and decrease full-cream milk selection through nudging. Therefore, it is possible that similar interventions in environments where full-cream milk selection is predominant might result in a different outcome.

## 5. Conclusions

While salience nudging is a potentially feasible intervention that could be easily implemented into workplace environments, the present study showed that over a three-month period a salience nudge was not effective for influencing milk selection in a workplace. Instead, nudging resulted in an overall increase in selection of both low-fat and full-cream milk in the first two weeks, before returning towards baseline milk selection levels. The initial increase in milk selection indicates the presence of a spill-over effect due to the nudge increasing the salience of milk. Caution should be used when nudging dietary choices, to ensure that spillover effects do not result in over consumption. These findings suggest that salience nudging may have limited application for assisting people in changing milk selection.
